# Lincomycin HCl-loaded nanoparticles: development, optimization, and incorporation into a nanogel for wound healing

**DOI:** 10.1039/d5ra08465b

**Published:** 2026-01-13

**Authors:** Aisha Sethi, Rabia Zaheer, Shazia Akram Ghumman, Asif Massud, Shazia Noureen, Ali Irfan, Mahwish Arshad, Muhammad Khawar Abbas, Mudassar Mazher, Yousef A. Bin Jardan

**Affiliations:** a Department of Pharmaceutics, Faculty of Pharmaceutical Sciences, Government College University Faisalabad-38000 Pakistan ayeshasethi786@gcuf.edu.pk rabiazaheer2018@gmail.com asifmassud@gmail.com; b College of Pharmacy, University of Sargodha Sargodha-40100 Pakistan shazia.akram@uos.edu.pk; c Institute of Chemistry, University of Sargodha Sargodha 40100 Pakistan shazianoureen11@gmail.com; d The Green Institute of Chemical, Biomedical and Environmental Sciences (GICBES) Lahore 54000 Pakistan draliirfan.ceo@gicbes.com raialiirfan@gmail.com; e Roy & Diana Vagelos Laboratories, Department of Chemistry, University of Pennsylvania Philadelphia Pennsylvania 19104-6323 USA maarshad@sas.upenn.edu; f Department of Medicine, Perelman School of Medicine, University of Pennsylvania Philadelphia Pennsylvania 19104 USA; g Department of Physics, Government College University Faisalabad 38000 Pakistan khawar.abbas@gcuf.edu.pk; h College of Pharmacy, University of Gujrat Pakistan mudassar@pharm.uchanab.edu.pk; i Department of Pharmaceutics, College of Pharmacy, King Saud University Riyadh 11451 Saudi Arabia ybinjardan@ksu.edu.sa

## Abstract

The present study focused on developing and evaluating lincomycin HCl (LCH)-loaded chitosan nanoparticles (CSNPs) incorporated into a nanogel system to improve wound healing. CSNPs were prepared *via* ionic gelation using sodium tripolyphosphate (STPP) as a cross-linker. The optimized formulation showed a mean particle size of 174.3 nm, a polydispersity index (PDI) of 0.267, a zeta potential of +29.4 mV, and a drug entrapment efficiency of 83.7%. FTIR, DSC, and XRD analyses confirmed successful drug encapsulation and stability with no chemical interactions. The formulation demonstrated sustained release (>75% in 24 hours) following non-Fickian kinetics. Antibacterial testing revealed improved efficacy against *Staphylococcus aureus* and *Escherichia coli* with inhibition zones of 45 mm ± 2.76 and 38 mm ± 2.15, respectively. *In vivo* wound healing studies in rats demonstrated almost complete wound closure within 14 days in the treated group, compared to significantly slower healing in the control group. These results show that the LCH–CSNP nanogel provides controlled drug release, effective antimicrobial action, and accelerated wound healing, highlighting its potential as a topical therapeutic platform.

## Abstract 2

The present study focused on developing and evaluating lincomycin HCl (LCH)-loaded chitosan nanoparticles (CSNPs) incorporated into a nanogel system to improve wound healing.

## Graphical abstract

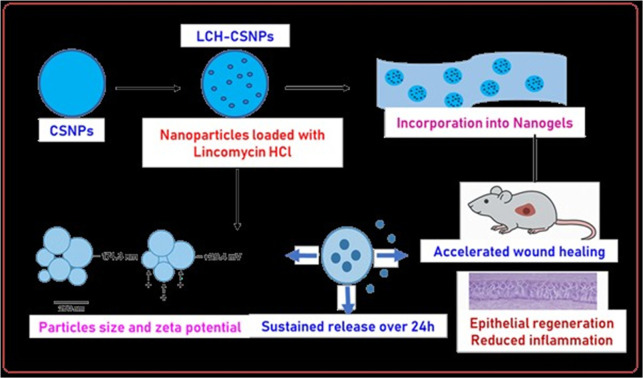

## 1. Introduction

Lincomycin HCl (LCH) is a bacteriostatic medication that inhibits the synthesis of susceptible bacteria. It can also eliminate high concentrations of bacteria present in the human body. Lincomycin belongs to class III of the biological classification system, characterized by low permeability and high solubility. It dissolves easily in water and is highly stable both in solution and in dry form.^1^ Lincosamides are a class of antibiotics that inhibit bacterial protein synthesis by disrupting multiple steps, from the initiation of amino acid monomers and the synthesis of aminoacyl-tRNAs to various phases of initiation, elongation, and termination of polypeptide chains on ribosomes.^2^ Lincomycin has effective antibacterial and anti-streptococcal properties. *In vitro* studies have confirmed that these antibiotics, even at low concentrations, reduce toxin release from producing strains.^3^

Bio-adhesive carriers like biodegradable polymer chitosan have been shown to increase residence time on mucosal tissue, improving treatment acceptance through better absorption and penetration.^1^ Recent research indicates that chitosan nanoparticles (CSNPs) with a mean particle size of 150–200 nm and a zeta potential of +30 mV possess optimal mucoadhesive properties, leading to a twofold increase in drug absorption and a threefold boost in therapeutic efficacy.^3^ CSNPs offer distinct advantages, including a large surface area (∼200 m^2^ g^−1^) and high zeta potential (+30 to +40 mV), making them versatile carriers for delivering drugs, vaccines, and genes. Notably, CSNPs have demonstrated 2- to 3-fold enhanced antibacterial activity against *E. coli* and *S. aureus* compared to regular chitosan, attributed to their unique nanoparticle characteristics.^5^

Additionally, CSNPs serve as effective drug carriers, facilitating controlled and sustained drug release, with studies showing up to 80% drug release over 24 hours.^6^ This property enhances drug solubility, stability, and efficacy while reducing toxicity.^7^ In critical situations, intravenous (IV) administration of CSNPs ensures rapid and complete drug absorption,^8^ offering a quick onset and desired therapeutic outcomes, but also a significant risk of adverse effects, particularly in critically ill patients. Shifting to alternative drug administration routes,^9^ such as the topical route, presents a safer option, provided it can be reliably predicted.^4^ Given that the skin serves as the body's largest organ, it holds great potential as a means of drug administration through transdermal applications, topical delivery, and skin-injected routes into underlying tissues.^5^ This approach involves delivering active ingredients across the skin for systemic distribution and is referred to as transdermal drug delivery.^6^

The skin serves as both a physical and chemical barrier against mechanical, UV, and microbial threats; breaches caused by accidents, burns, or diseases like ulcers pose risks.^7^ Wounds, categorized as acute or chronic, may face complications influenced by age, diet, infections, and medications.^8^ Chronic wounds, prevalent in conditions like diabetes, demand efficient healing therapies. Despite the availability of conventional wound dressings and topical antimicrobial agents, their therapeutic efficacy is often limited by poor drug retention at the wound site, inadequate penetration, frequent application requirements, and the risk of systemic side effects.^9^ These limitations have driven increasing interest in advanced wound-healing strategies that can provide sustained drug release, maintain a moist wound environment, prevent microbial colonization, and actively promote tissue regeneration. Biopolymer-based nanocarriers, particularly chitosan-based nanoparticulate systems, have attracted considerable attention due to their biocompatibility, biodegradability, intrinsic antimicrobial activity, and ability to enhance wound repair.^10^

In our current studies, we employed the ionic gelation technique, utilizing sodium tripolyphosphate (STPP) as a cross-linker to create chitosan (CS) nanoparticles (NPs) loaded with lincomycin HCl (LCH). The novelty of this approach lies in optimizing the CS/STPP molar ratio to achieve tailored NP sizes and drug release profiles, thereby enhancing the antimicrobial efficacy of LCH. We hypothesize that the incorporation of LCH into CSNPs *via* ionic gelation will not only improve the drug's solubility and stability but also facilitate sustained release and targeted delivery to the wound site, ultimately leading to accelerated wound healing and reduced bacterial load. The resultant NPs' mean diameter and drug release characteristics are significantly influenced by the CS/STPP molar ratio, which was carefully optimized in this study. The antimicrobial characteristics of LCH–CSNPs were evaluated using a wound-induced injury model in rats, with a focus on assessing the NPs' ability to promote wound closure and tissue regeneration.

## 2. Materials and methods

### 2.1. Material

Lincomycin HCl was a gift sample from Majestic Pharma. Low molecular weight chitosan powder (biological extract, (C_6_H_11_NO_4_)_*n*_), acetic acid anhydride, polyethylene glycol 400 (PEG400), sodium tripolyphosphate (STPP), Carbopol 940 obtained from Sigma Aldrich. Distilled water, and ethanol purchased from the local scientific store. All chemicals used were of analytical grade.

### 2.2. Preparation of CSNPs

The preparation of CSNPs followed the ionic gelation technique, incorporating slight modifications to the previously described method.^11^ Chitosan (CS) solutions with various concentrations of 1, 2, and 3 mg mL^−1^ were dissolved in 50 mL of 1% (v/v) acetic acid. The pH was adjusted to 4.7 using 2 M NaOH. Sodium tripolyphosphate (STPP) solution was dissolved in 50 mL of deionized water at a concentration of 1 mg mL^−1^, and pH was adjusted accordingly. CS and STPP solutions were filtered through a 0.45 µm syringe filter, respectively, to ensure removal of insoluble materials. Subsequently, the STPP solution was added dropwise into the CS solution under continuous magnetic stirring at room temperature, resulting in the formation of an opalescent colloidal suspension indicative of nanoparticle formation. Following this, the solution underwent an aging period lasting 12 to 18 hours. Afterward, the chitosan nanoparticles (CSNPs) solution was subjected to centrifugation and thoroughly washed with phosphate-buffered saline at a pH of 7.4, followed by additional rinsing with Milli-Q water to remove any excess acetic acid and residual by-products. The pH of the CSNP solution was subsequently adjusted to 7.0 after this washing process.

### 2.3. Preparation of LCH–CSNPs

The CSNPs were washed and dispersed in 10 mL of Milli-Q water; LCH was loaded into CSNPs at a concentration of 0.5 µg mL^−1^, and antibiotic functionalization was performed at room temperature (300 K) under dark conditions, followed by continuous stirring of the LCH–CSNPs mixture for 24 h. Notably, upon antibiotic addition, the reaction became endothermic within a few hours, with the temperature recorded at 297 ± 2 K, suggesting a spontaneous interaction between the drug and the nanoparticle surface. To remove unbound antibiotics, the mixture was centrifuged three times, and the resulting precipitates were redispersed in Milli-Q water using an ultrasonic homogenizer. These functionalized nanoparticles were subsequently used for further analyses.^12^

### 2.4. Characterization techniques

#### 2.4.1. Fourier transform infrared (FTIR)

Fourier transform infrared (FTIR) spectroscopy was used to assess the veracity of the functional groups and stoichiometric features of the deposited coating. Measurements were made using a Shimadzu 8400S spectrometer, in transmission mode, operating in the 5000–500 cm^−1^ range. Individual samples were analyzed and recorded.^13^

#### 2.4.2. Differential scanning calorimetry (DSC)

Thermal analysis was employed to study the response of various substances toward temperature, including CS, LCH, blank NPs, and LCH–CSNPs. This analysis was conducted using a Differential Scanning Calorimeter (DSC) from TA Instruments (Model 302, Germany).^14^

#### 2.4.3. X-ray diffraction (XRD)

X-ray diffraction (XRD) analysis was performed using a Rigaku MiniFlex X-ray Diffractometer with Cu Kα radiation (*λ* = 1.5418 Å). The samples, finely ground into powder, were placed on a zero-background silicon sample holder. The XRD patterns, presented as diffractograms with 2*θ* angles on the *x*-axis and diffracted intensity on the *y*-axis, provided a comprehensive view of the crystalline structures.^15^ Data was collected in the 2*θ* range of 5° to 90° with a step size of 0.02° and a scanning speed of 2° per minute, with each data point collected for 1200 seconds to enhance quality. The XRD data were analyzed using Jade XRD analysis software, which facilitated peak fitting and background subtraction. Pattern indexing was done by comparing with the International Centre for Diffraction Data (ICDD) databases. The instrument's accuracy was ensured through calibration with a standard silicon powder sample, and experiments were conducted in a lead-lined cabinet with appropriate safety gear. Control experiments with standard reference materials validated the instrument's performance and data quality, and reproducibility was confirmed through multiple experiments on the same samples, showing high consistency.

#### 2.4.4. Entrapment efficiency and drug content

The entrapment efficiency and drug content of freshly prepared nano-formulation was examined by centrifuging 1 mL of the formulation at 6000 rpm for 30 minutes in a nano centrifuge machine.^16^ The resulting supernatant, the unencapsulated (free) drug, was evaluated using a UV spectrophotometer, and entrapment efficiency (EE%) and drug content (DC%) were calculated by using the following equations.
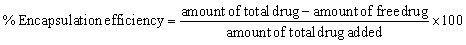

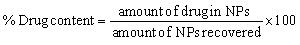


#### 2.4.5. Particle size and zeta potential

Particle size and zeta potential of the nano suspension diluted in distilled water were examined by dynamic light scattering (DLS) using Malvern nanoZS90, UK.^17^

#### 2.4.6. Morphology of LCH–CSNPs

LCH–CSNPs were subjected to SEM analysis on a scanning electron microscope (JEOL 1.1, JSM-5910). Samples are mounted on the holder and layered with gold film to provide an even conductivity surface and smooth analysis. The system was operated under vacuum at different magnifications.^18^

#### 2.4.7. Identification of culture

A total of 20 suspected *Staphylococcus aureus* (*S. aureus*) cultures (two from each sample) and 20 suspected *E. coli* cultures (two from each sample) were randomly selected for further analysis. Traditional identification methods were employed, including assessment of colony morphology on selective media, Gram staining, and biochemical characterization, followed the procedure described in Bergey's Manual of Systematic Bacteriology. The antimicrobial activity of CSNPs and LCH–CSNPs was evaluated against *Staphylococcus aureus* (*S. aureus*), which is a Gram-positive bacterium, and *Escherichia coli* (*E. coli*), a Gram-negative bacterium. Bacterial sensitivity to antibiotics was determined using the well diffusion method. LCH was tested alone and in combination with CSNPs to assess the enhanced antibacterial effect of the nanoparticle-antibiotic formulation.^19^

### 2.5. *In vitro* release profiles

In a comparative *in vitro* release, we investigated the release of a drug from chitosan nanoparticles under varying pH conditions (1.2, 7.4) using a phosphate buffer solution (PBS). First, we prepared chitosan nanoparticles and formed a suspension using 5.0 mL of PBS. Subsequently, we enclosed this nanoparticle suspension within a dialysis membrane with a molecular weight cutoff of 12 kDa, securely tying both ends of the membrane. This membrane, containing the nanoparticle suspension, was immersed in a cell containing 100 mL of PBS and placed in a shaker incubator (Farazma, Iran) set at a constant temperature of 37 °C. The shaker operated at 25 rpm to ensure thorough mixing and agitation. At specified intervals, we withdrew 5 mL samples from the cell, representing the contents of the receiving compartment. After each sampling, we replenished the cell with an equal volume of fresh pre-warmed PBS to maintain a constant sink condition and prevent saturation effects. To determine the drug concentration in the collected samples, we likely used a spectrophotometer or similar analytical equipment to measure absorbance at a wavelength of 270 nm. These steps enabled us to calculate the drug release at each time point based on the concentration measurements. The calculated values were then utilized to construct a cumulative release curve, illustrating the drug release profile over time.^20^

### 2.6. Acute toxicity studies of LCH–CSNPs

In the designated animal facility at Government College University, Faisalabad, Pakistan, adult male Wistar rats (weighing between 150–200 g and aged 10–12 weeks) were housed in well-ventilated polypropylene cages under standard conditions (maintaining a temperature of 25 ± 2 °C, a 12 hour light–dark cycle, and humidity ranging from 55–60%). The rats were provided with standard commercial pelleted rat feed and had access to water *ad libitum*. Specially designed cages were employed to separate the urine and feces of the study animals. The bedding of the animal cages was refreshed every 48 hours.^21^ Before the commencement of the study, the rats underwent a week-long acclimatization period and were then randomly divided into six groups, each consisting of six rats (*n* = 6). The oral route was utilized for the toxicity assessment.

### 2.7. LCH–CSNPs integrated into a nanogel for wound healing applications

Carbopol 940 was used as the gelling agent in this formulation. A 1% aqueous solution of Carbopol 940 was prepared and stirred continuously at 800 rpm for 1 hour using a magnetic stirrer. The pH of the solution was then adjusted to 6.2–6.8 by adding 0.05% triethanolamine. Dried LCH–CSNPs, equivalent to 1% w/w of lincomycin in the gel, were incorporated into the Carbopol solution under continuous stirring to ensure uniform distribution. Carbopol was selected for topical application due to its non-irritating and non-toxic nature, stability across a wide temperature range, and absence of hypersensitivity reactions.^22^ An *in vivo* study on wound healing was conducted using adult male Wistar rats, adhering to ethical guidelines and institutional review board approval (Ref. No. 39A24GCUF/ERC).

All *in vivo* experiments were conducted in strict accordance with internationally accepted ethical standards for animal experimentation. The study protocol was reviewed and approved by the Institutional Ethical Review Committee (IERC) of Government College University, Faisalabad (Approval No. 39A24GCUF/ERC). Adult Wistar rats (non-pregnant, nulliparous females), weighing 180–220 g, were used in this study. Animals were housed under standard laboratory conditions (temperature 22 ± 2 °C, relative humidity 55 ± 10%, 12 h light/dark cycle) with free access to standard pellet diet and water *ad libitum*.

Wound induction and treatment procedures were performed under appropriate anesthesia to minimize pain and distress, and all efforts were made to reduce animal suffering in accordance the experimental procedures were conducted in compliance with relevant OECD recommendations and internationally accepted laboratory animal care guidelines. A standardized infected excision wound model was used to evaluate *in vivo* wound-healing performance. Two full-thickness wounds, each with a diameter of 5 mm, were then created on the rats' backs using a biopsy punch. The wound was infected with an inoculum containing *S. aureus* and *E. coli.* The respective treatments were topically applied to the wound areas once daily. Wound healing progression was monitored on days 0, 3, 5, 7, 10, and 12 by capturing photographic images of the wound sites. The healing process was evaluated qualitatively based on visible wound closure, reduction in wound area, and scab formation.^23^

### 2.8. Statistical analysis

Statistical analysis was performed on experimental data using one-way and two-way ANOVA with Tukey multiple comparisons and Bonferroni post-test, respectively, with significant results at *p* < 0.05 (GraphPad Prism 5 software). Results were presented as mean ± SEM (*n* = 5).

## 3. Results and discussions

### 3.1. Fourier transform infrared spectroscopy (FTIR)

**Fig. 1:**
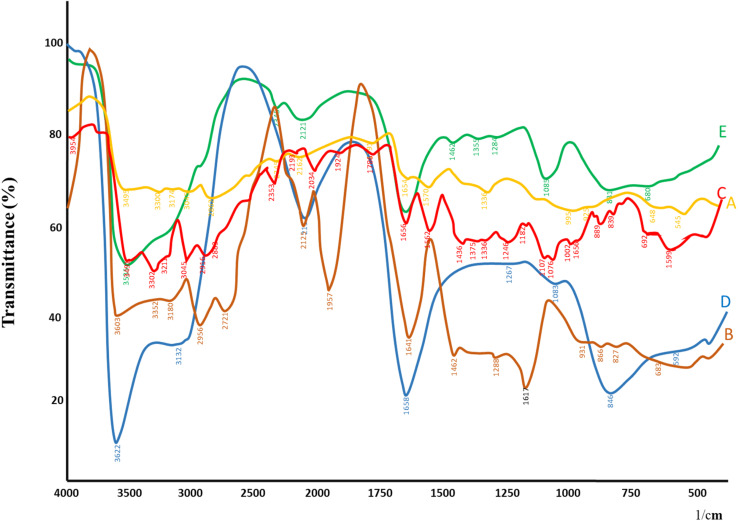
FTIR spectra of the (A) CS, (B) STPP, (C) LCH, (D) blank CSNPs, (E) LCH–CSNPs.

FTIR spectroscopy was employed to investigate potential interactions among LCH, CS, STPP, and the nano-formulation. The pure drug (LCH) exhibited characteristic peaks at 3449 cm^−1^ (N–H/O–H stretching), 1636 cm^−1^ (C=O stretching of amide), and 1020–1200 cm^−1^ (C–N and C–O stretching vibrations), confirming its structural integrity. Chitosan displayed prominent absorption bands at 3449 cm^−1^ due to overlapping O–H and N–H stretching vibrations, along with peaks at 1640 cm^−1^ and 1380 cm^−1^ corresponding to amide I and C–H bending, respectively.^24^ STPP showed characteristic phosphate stretching vibrations between 900–1250 cm^−1^, primarily attributed to P=O and P–O–P bonds.^25^ The blank formulation, composed of chitosan cross-linked with STPP, demonstrated notable peak shifts and broadening, particularly in the 3400–1600 cm^−1^ region, indicating successful ionic gelation and crosslinking.^26^ In the FTIR spectrum of the drug-loaded formulation as depicted in Fig. 1, the presence of lincomycin's characteristic peaks, though shifted or attenuated, suggested successful drug entrapment. The shift and broadening of the –OH and –NH stretching bands around 3449 cm^−1^, and the overlapping of phosphate and amide bands in the 1630–1250 cm^−1^ range, confirm intermolecular hydrogen bonding and electrostatic interactions between the drug and polymeric matrix. Importantly, no new peaks were observed, suggesting the absence of any chemical incompatibility or covalent bond formation, implying that the drug was physically encapsulated within the polymeric network.^27^ These findings validated the successful incorporation of LCH into CSNPs, supported the structural stability and compatibility of components within the developed formulation.

### 3.2. Differential scanning calorimetry (DSC)

**Fig. 2:**
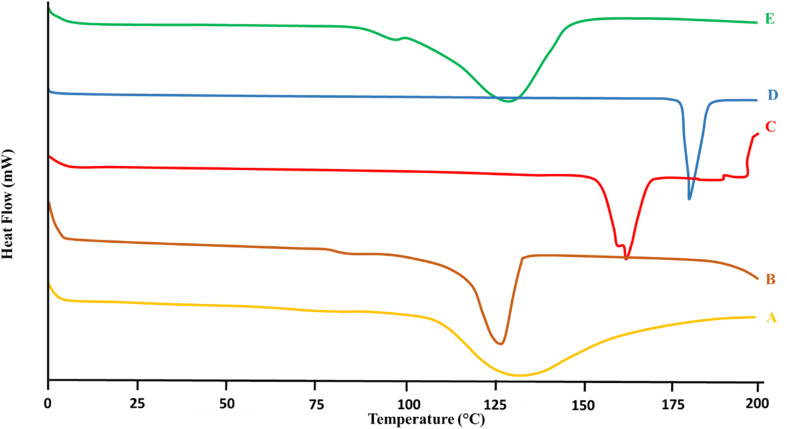
DSC of the (A) LCH, (B) CS, (C) STPP, (D) blank CSNPs, (E) LCH–CSNPs.

DSC was performed to evaluate the thermal characteristics and physical state of LCH in the developed nanoparticles as displayed in Fig. 2. The thermogram of pure LCH showed a sharp endothermic peak at 191.6 °C, corresponding to its melting point, confirming its crystalline nature.^1,28^ CS (polymer) displayed a broad endothermic peak around 105–130 °C, which was attributed to the loss of bound moisture and glass transition behavior.^29^ The cross-linker (STPP) showed a thermal event at approximately 140.2 °C, likely due to structural relaxation or dehydration.^30^ In the blank CSNPs, a broad thermal transition was observed between 120–150 °C, indicating successful cross-linking between chitosan and STPP, forming a stable polymeric network.^11^ Notably, the LCH–CSNPs exhibited no sharp melting peak near 191.6 °C, indicating the absence of crystalline LCH. Instead, a broad and diffused thermal event was observed around 140–160 °C, suggesting that the drug was molecularly dispersed or converted to an amorphous state within the nanoparticle matrix.^31^ The disappearance or shift of the drug's melting endotherm confirmed successful encapsulation and possible intermolecular interactions between LCH and the CS–STPP matrix. This amorphous conversion can potentially improve the solubility, dissolution rate, and bioavailability of the drug.

### 3.3. X-ray diffraction (XRD)

**Fig. 3:**
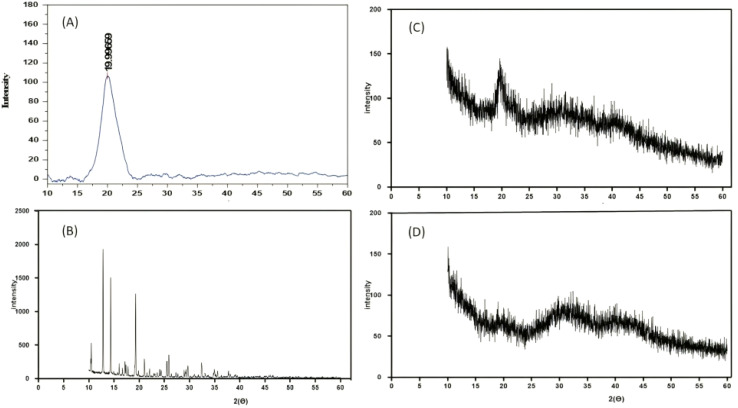
XRD of the (A) CS, (B) LCH, (C) blank CSNPs, and (D) LCH–CSNPs.

XRD analysis was performed to investigate the crystalline or amorphous nature of LCH in its pure form and within the developed nanoparticle formulation. The XRD pattern of pure LCH (red) showed multiple sharp, intense peaks in the 2*θ* range of 10° to 35°, with prominent peaks around 2*θ* = 12.8°, 17.4°, 20.1°, 25.7°, and 27.6°, indicating its highly crystalline nature as demonstrated in Fig. 3. These peaks are characteristic of a well-ordered molecular lattice and confirm that the raw drug exists in a crystalline form. In contrast, chitosan (black) exhibited a broad, diffused hump centered around 2*θ* = 20°, which is typical of semi-crystalline polymers. This broad peak indicates limited molecular order due to the flexible backbone and intermolecular hydrogen bonding in chitosan. The blank CSNPs (yellow) showed a similarly broad halo without any sharp peaks, indicating the formation of an amorphous cross-linked network. The absence of sharp crystalline peaks suggests that the ionic interaction between chitosan and STPP disrupted any residual crystalline regions in chitosan. Importantly, the XRD pattern of LCH–CSNPs (blue) showed a complete absence of the characteristic crystalline peaks of LCH, indicating that the drug was either molecularly dispersed or transformed into an amorphous form during nanoparticle formation. The pattern closely resembles that of the blank nanoparticles, confirming that drug loading did not alter the amorphous nature of the matrix.^32^ The loss of crystalline peaks in the drug-loaded formulation supports findings from DSC, where the melting endotherm of LCH was also absent. Together, these results confirmed the successful encapsulation of LCH in an amorphous or non-crystalline state, which was advantageous for enhancing solubility, dissolution rate, and bioavailability of the drug.

### 3.4. Particle size and PDI

The nanoparticulate systems (F1–F3) were developed using the ionic gelation method, where CS served as the cationic polymer and STPP as the polyanionic cross-linker, with LCH incorporated as the model drug. LCH–CSNPs exhibited an entrapment efficiency ranging from 68.49 ± 0.55% to 85.72 ± 0.41%. The drug content was observed between 85.52 ± 0.76% and 94.77 ± 0.39%. The influence of varying chitosan concentrations (50, 100, and 150 mg) at a constant STPP level (1 mg) on particle size, polydispersity index (PDI), and zeta potential is presented in Table 1.

**Table 1:** Percentage (%) E.E, and % D.C of LCH–CSNPs in mean ± standard deviation (S.D)/*n* = 3

Formulation	CS^a^ : STPP^b^ (mass ratio)	% E.E^c^	% D.C^d^
F1	1 : 1	79.43 ± 0.11	85.52 ± 0.76
F2	2 : 1	85.72 ± 0.41	94.77 ± 0.39
F3	3 : 1	68.49 ± 0.55	78.21 ± 0.67

a CS: chitosan.

b STPP: sodium tripolyphosphate.

c E.E: entrapment efficiency.

d D.C: drug content.

**Fig. 4:**
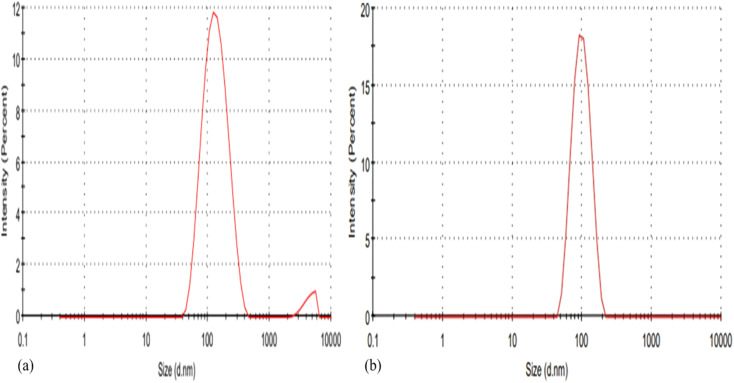
Zeta size of the (a) CSNPs and (b) LCH–CSNPs.

The particle size of blank CSNPs was (265.0 nm), and LCH–CSNPs was (152.4 nm) as shown in Fig. 4. This reduction in size can be explained by the electrostatic attraction between positively charged chitosan and lincomycin HCl, which promotes tighter polymer chain arrangement and limits particle growth during ionic gelation. This interaction likely suppresses nanoparticle swelling and aggregation, yielding smaller and more uniform particles.^33^

### 3.5. Zeta potential

**Fig. 5:**
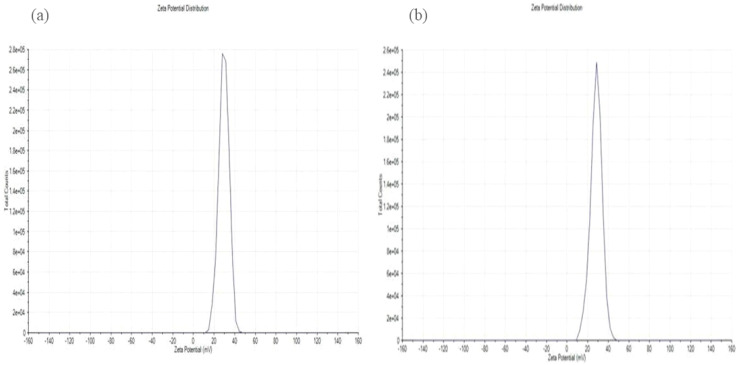
Zeta potential of the (a) CSNPs and (b) LCH–CSNPs.

Zeta potential is a key indicator of the surface charge and colloidal stability of nanoparticle formulations. The zeta potential of the LCH–CSNPs was found to be +29.4 mV, which indicated a moderately high positive surface charge as showed in Fig. 5. This positive charge can be attributed to the presence of chitosan, a cationic polymer, which imparts a positive surface potential due to its protonated amino groups (–NH_3_^+^) under acidic or slightly neutral pH conditions. The observed zeta potential value suggests that electrostatic repulsion between particles is sufficient to prevent aggregation, contributing to the physical stability of the nanoparticle suspension. Generally, zeta potential values greater than ±25 mV are considered to confer good stability to colloidal systems by minimizing particle–particle interactions. The blank CSNPs have a zeta potential of +28.2 ± 5.54 mV, which is characteristic of chitosan due to the protonated –NH_3_^+^ groups on its surface and shows successful formation of CSNPs, confirming that chitosan is effectively present on the nanoparticle surface. The LCH–CSNPs exhibited the measured value of +29.4 mV, which suggest electrostatic stabilization of the nanoparticles and supports their suitability for topical wound-healing applications. Furthermore, the positive surface charge may enhance mucoadhesive properties and promote interaction with negatively charged biological membranes, potentially improving cellular uptake and therapeutic efficacy of the encapsulated drug.^34^

### 3.6. SEM analysis

**Fig. 6:**
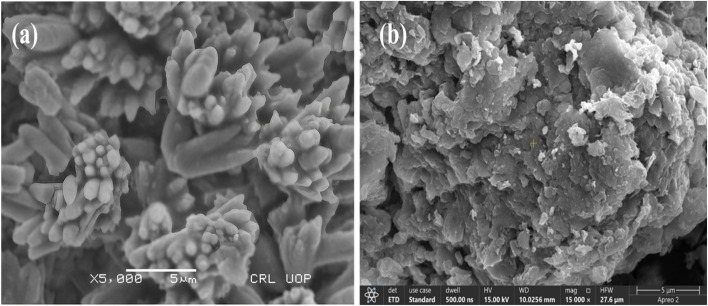
SEM image of (a) LCH–CSNPs (5 µm) and (b) LCH–CSNPs loaded NGs (5 µm).

The SEM analysis of LCH–CSNPs (Fig. 6a) revealed clustered, rod-like structures with a rough surface morphology, indicating successful ionic gelation of chitosan with STPP and effective drug entrapment within the polymeric matrix as indicated in Fig. 6. The nanoparticles exhibited semi-crystalline domains and a high surface area, which are favorable for enhanced drug release kinetics^35^ (Table 2). In contrast, the SEM image of LCH–CSNGs (Fig. 6b) displayed an amorphous, sponge-like, and porous architecture with irregular folds and embedded nanoparticles, confirming the formation of a hydrogel network. This porous morphology is advantageous for swelling, bio-adhesion, and controlled release of the drug, providing a sustained therapeutic effect compared to the relatively faster release expected from nanoparticles. Collectively, the SEM results demonstrate that while nanoparticles ensure high drug loading and initial release, incorporation into nanogels offered a porous matrix that enhanced applicability for localized wound healing.^36^

**Table 2:** Models for drug release kinetics

Samples	pH	Models for drug release kinetics				
		Zero order	First order	Higuchi	Korsmeyer–Peppas	
		*R* ^2^	*R* ^2^	*R* ^2^	*R* ^2^	N
F1	1.2	0.8823	0.8849	0.8094	0.8933	0.843
	7.4	0.9285	0.9611	0.8950	0.9557	0.769
F2	1.2	0.9693	0.9696	0.7967	0.9743	1.129
	7.4	0.8815	0.9465	0.8842	0.9273	0.715
F3	1.2	0.8932	0.8960	0.8074	0.9006	0.868
	7.4	0.8485	0.9502	0.8902	0.9184	0.665
Drug	1.2	0.1921	0.2135	0.8077	0.9115	0.299
	7.4	−0.5738	0.9249	0.4269	0.8638	0.148

### 3.7. Release studies

**Fig. 7:**
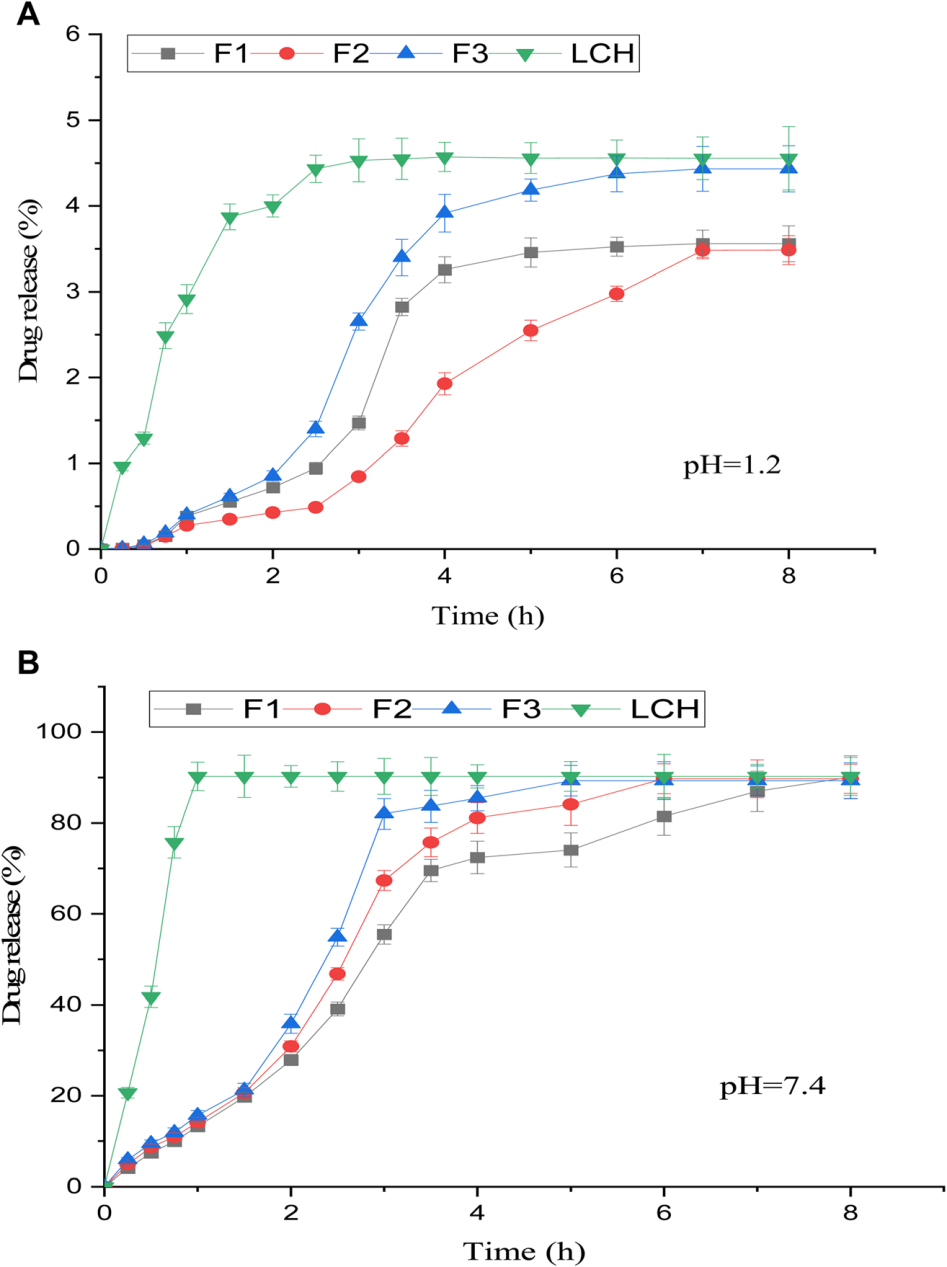
(A) *In vitro* drug release profile of LCH–CSNPs, (F1, F2, F3, and LCH) at 37 °C; at pH 1.2. Significant difference between formulations (<0.05). (B) *In vitro* drug release profile of LCH–CSNPs (F1, F2, F3, and LCH) at 37 °C, at pH 7.4 for 10 hours. Significant difference between formulations (<0.05).

The *in vitro* release behavior of LCH–CSNPs (F1, F2, F3) was systematically evaluated at two physiological pH conditions, pH 1.2 (simulating the gastric environment) and pH 7.4 (simulating the intestinal environment), and compared with the release profile of the free LCH drug as displayed in Fig. 7A and B, respectively. At pH 1.2, the free drug exhibited an immediate and almost complete release, reaching a plateau within 2 hours, owing to its high aqueous solubility and absence of any diffusion barrier. This rapid release indicated that unencapsulated LCH cannot withstand the acidic gastric environment and is likely to be absorbed prematurely, possibly leading to suboptimal therapeutic efficacy or side effects. In contrast, the nanoparticle formulations demonstrated a significantly delayed and controlled release at this acidic pH. F1 released approximately 65–70% of the drug within 5 hours, while F2 and F3 displayed even more prolonged release, with F3 showing the slowest release rate. This controlled behavior was attributed to the protonation of chitosan at low pH, which led to polymer swelling and formation of a gel-like barrier that restricts the diffusion of the encapsulated drug. Among the formulations, F3 exhibited the highest resistance to acidic degradation, possibly due to a denser polymer network or higher crosslinking, making it ideal for minimizing drug loss in the stomach.^1^

At pH 7.4, the release profiles changed drastically. The free drug again showed a burst release, with more than 95% released within the first 30 minutes, indicating poor retention and rapid dissolution in intestinal fluids. In contrast, the nanoparticle formulations exhibited a sustained and gradual release, with F1 releasing approximately 85% of the drug by 6 hours, while F2 and F3 achieved near-complete release (90–100%) within the same period, albeit in a more controlled manner. This difference in behavior was due to the deprotonation of chitosan at higher pH, leading to polymer relaxation and erosion, which facilitates drug diffusion. Notably, F3 maintained the most sustained release even at pH 7.4, suggesting a robust formulation capable of protecting the drug during its passage through the stomach and ensuring targeted and extended delivery in the intestine.^37^

In summary, the comparative release profiles demonstrated that while free LCH dissolves rapidly at both pH levels, it lacks controlled delivery and may be unsuitable for prolonged therapeutic action. In contrast, chitosan nanoparticle-based formulations, particularly F3, offer pH-sensitive, controlled release properties, protecting the drug in the acidic stomach and promoting sustained release in the intestinal environment. This makes them highly promising carriers for oral delivery of LCH, enhancing its therapeutic window, reducing dosing frequency, and potentially improving patient compliance.

### 3.8. Antimicrobial activity of CSNPs and LCH–CSNPs

**Fig. 8:**
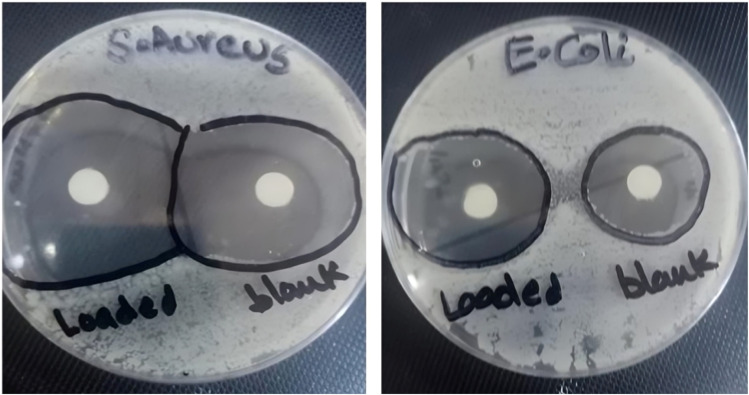
Zone of inhibition of blank CSNPs and loaded LCH–CSNPs with (a) *S. aureus*, (b) *E. coli*.

The antimicrobial activity of blank CSNPs and LCH–CSNPs was assessed against Gram-positive bacteria, *Staphylococcus aureus*, and Gram-negative bacteria, *Escherichia coli*, using the agar well plate diffusion method.^35^ The blank CSNPs showed a small inhibition zone of 3.7 mm ± 0.63 against *S. aureus* and of 2.9 mm ± 0.85 against *E. coli*, confirming that chitosan itself possesses mild intrinsic antibacterial properties due to its cationic nature, which disrupts bacterial cell membranes as displayed in Fig. 8. In contrast, when LCH–CSNPs were applied, a substantial increase in the inhibition zone was observed. When tested against *S. aureus*, the inhibition zone expanded from 3.7 mm to 45 mm ± 2.76 with the introduction of LCH–CSNPs. This enhancement emphasized the heightened antimicrobial effect against *S. aureus* resulting from the integration of LCH with CSNPs. Similarly, in the case of *Escherichia coli*, we observed an increase in the inhibition zone, growing from 2.9 mm to 38 mm ± 2.15 when LCH was incorporated. This remarkable enhancement indicates a synergistic antimicrobial effect between LSH and CSNPs, suggesting that drug loading into the CSNP matrix amplified antibacterial potency by promoting sustained release and efficient bacterial membrane interaction.^35,38^

### 3.9. Acute toxicity studies of LCH–CSNPs

Assessment of social adjustments and weight vacillations in rodents from control and LCH–CSNPs-treated groups during intense toxicity examination. A single oral dose of 100 mg kg^−1^ of the liquid dispersion was administered. Eminently, no recognizable changes were identified in social or mental boundaries, and there were no announced occasions of mortality during the 14 day observation period, as mentioned in Table 3. Such perceptions give vigorous proof supporting the case that the LD_50_ of LCH surpasses 100 mg kg^−1^. Significantly, there was no way to see any modifications in conduct or mental boundaries, and no instances of mortality were accounted for throughout the whole 14 day observation period, as carefully kept in Table 3. Further examination of body weight uncovered a genuinely immaterial contrast between the benchmark group and the anti-infection treated group, as displayed in Table 4. These discoveries unequivocally supported the statement that the LD_50_ of lincomycin HCl surpasses 100 mg kg^−1^.^39^

**Table 3:** Change in parameters in the acute toxicity study

Parameters	Observations of the control, blank and LCH–CSNPs-treated group														
	30 minutes			4 h			24 h			7 days			14 days		
	Cl	Bl	NPs	Cl	Bl	NPs	Cl	Bl	NPs	Cl	Bl	NPs	Cl	Bl	NPs
Eye color	N	N	N	N	N	N	N	N	N	N	N	N	N	N	N
Pupil size	N	N	N	N	N	N	N	N	N	N	N	N	N	N	N
Salivation	N	N	N	N	N	N	N	N	N	N	N	N	N	N	N
Skin	N	N	N	N	N	N	N	N	N	N	N	N	N	N	N
Fur	N	N	N	N	N	N	N	N	N	N	N	N	N	N	N
Urine color	N	N	N	N	N	N	N	N	N	N	N	N	N	N	N
Feces consistency	N	N	N	N	N	N	N	N	N	N	N	N	N	N	N
Convulsions & tremors	N	N	N	N	N	N	N	N	N	N	N	N	N	N	N
Itching	N	N	N	N	N	N	N	N	N	N	N	N	N	N	N
Coma	N	N	N	N	N	N	N	N	N	N	N	N	N	N	N
Sleep	N	N	N	N	N	N	N	N	N	N	N	N	N	N	N
Somatomotor activity & behavior pattern	N	N	N	N	N	N	N	N	N	N	N	N	N	N	N

**Table 4:** (100 mg kg^−1^) on body weight (g) of rats in acute toxicity study

Days	Body weight (g)	
	Control	Treatment (100 mg kg^−1^)
1	163.6 ± 1.36	177.2 ± 1.40^ns^
7	171.2 ± 1.98	174.2 ± 0.86^ns^
14	192.4 ± 1.60	196.4 ± 1.03^ns^

### 3.10. Impact on hematological and biochemical parameters in the course of an acute toxicity investigation

In the acute toxicity testing, a thorough examination of Complete Blood Count (CBC) was conducted, comparing the control group with the group treated with LCH–CSNPs. The findings revealed no significant changes in most parameters, except for notable variation in platelet count and white blood cell count, as delineated in Table 5. It is crucial to note that despite this variance, the platelet count remained within the normal range, reinforcing the conclusion that the tested LCH–CSNPs exhibited a favorable safety profile. An expansion in white blood cell (WBC) count commonly showed activation of the resistance framework because of contamination brought by *E. coli* and *S. aureus*.^40^ This disparity offered help for the attestation that the anti-infection has antimicrobial action, subsequently offering significant insight into the general security evaluation.

**Table 5:** Effect of LCH–CSNPs (100 mg kg^−1^) on hematological parameters in an acute toxicity study

Parameters	Units	Normal control	Blank treated	LCH–CSNPs-treated
Haemoglobin	g dL^−1^	11.4 ± 0.298	12.78 ± 0.306^ns^	12.6 ± 0.51^ns^
TLC	×10^9^ L^−1^	11.0 ± 0.70	11.9 ± 0.57^ns^	12.0 ± 0.55^ns^
Total RBC	×10^12^ L^−1^	4.20 ± 0.19	6.54 ± 0.16^ns^	7.58 ± 0.32^ns^
HCT (PCV)	%	40.5 ± 0.58	38.8 ± 0.86^ns^	40.6 ± 0.68^ns^
MCV	Fl	64.0 ± 1.82	52.2 ± 1.77^ns^	53.6 ± 1.34^ns^
MCH	Pg	14.6 ± 1.20	19.8 ± 0.70^ns^	16.6 ± 0.65^ns^
MCHC	%	35.6 ± 0.65	35.2 ± 1.00^ns^	31.0 ± 1.30^ns^
Platelets	×10^3^ µL^−1^	561.8 ± 5.81	614.4 ± 1.6***	971 ± 2.0***
Neutrophils	%	36.9 ± 2.44	44.8 ± 1.29^ns^	17 ± 1.79^ns^
Lymphocytes	%	23.8 ± 1.65	39.6 ± 1.22^ns^	79 ± 1.20^ns^
Monocytes	%	2.74 ± 0.73	3.2 ± 0.81^ns^	3.0 ± 1.71^ns^
Eosinophils	%	1.45 ± 0.31	1.32 ± 0.78	1.0 ± 0.64
Total WBCs	10^3^ µL^−1^	14.22 ± 0.29	19.18 ± 0.65	17 ± 1.56

**Fig. 9:**
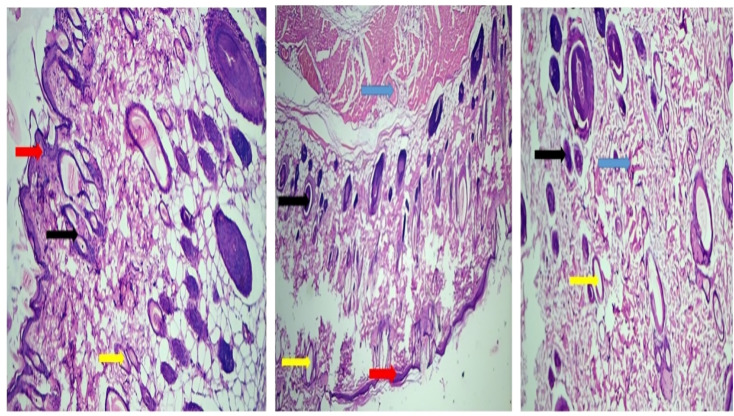
Histopathological examination revealed the presence of macrophages (black arrows), monocytes (yellow arrows), intact epidermal layer (red arrows), and erythrocytes (blue arrows). The treated group (R1) showed well-organized epidermis with increased immune cell infiltration, indicating active tissue regeneration and wound healing.

The provided data from the table indicates that within the groups provided with treatment, there were observed elevations in blood urea levels accompanied by lower creatinine levels. Alkaline phosphatase, bilirubin, protein, albumin, and globulin levels were found to be within the normal range, as observed in Table 6. However, there were elevated levels of AST (Aspartate Aminotransferase) and ALT (Alanine Aminotransferase) enzymes noted. This pattern suggests potential renal involvement, as evidenced by elevated blood urea levels coupled with lower creatinine levels, indicative of altered kidney function.^41^ Meanwhile, the normal range of alkaline phosphatase, bilirubin, protein, albumin, and globulin suggests intact liver and hepatic function. The elevation in AST and ALT enzymes indicates liver injury or damage, which could be associated with the treatment administered, as determined in Fig. 9.

**Table 6:** Effect of LCH–CSNPs loaded nanogels (2000 mg kg^−1^) on liver and kidney function test in acute toxicity study

Parameters	Units	Control	Blank treated	LCH–CSNPs-treated
Bilirubin	mg dL^−1^	0.20 ± 0.01	0.33 ± 0.017^ns^	0.3 ± 0.023
ALT	µ L^−1^	48.0 ± 1.65	76.0 ± 2.43*	61 ± 2.87
AST	µ L^−1^	241 ± 1.80	331 ± 2.54^ns^	272 ± 2.64
Alkaline phosphatase	µ L^−1^	243 ± 3.03	298 ± 3.35^ns^	341 ± 3.84
Protein	g dL^−1^	7.5 ± 0.23	6.7 ± 0.19^ns^	6.9 ± 0.25
Albumin	g dL^−1^	4.3 ± 0.15	3.7 ± 0.09^ns^	4.0 ± 0.04
Globulin	g dL^−1^	3.2 ± 0.122	3.0 ± 0.16^ns^	2.9 ± 0.21
Blood urea	mg dL^−1^	53.0 ± 1.41	49 ± 1.65^ns^	46 ± 1.92
Serum creatinine	mg dL^−1^	0.42 ± 0.034	0.33 ± 0.025^ns^	0.31 ± 0.043

### 3.11. LCH–CSNPs–NGs wound healing applications

The photographic representation of wound healing over 12 days clearly demonstrates the comparative efficacy of different treatments in promoting tissue regeneration and repair. In the control group, the wounds exhibited minimal healing, characterized by persistent scabbing and visible inflammation throughout the observation period. The lack of active treatment resulted in slower epithelialization and delayed closure, highlighting the natural but inefficient healing process.

In the BS (blank CSNPs infected with *S. aureus*) and BE (blank CSNPs infected with *E. coli*) groups, partial wound contraction was observed by day 12. However, these groups showed moderate healing with visible scab formation and incomplete epithelialization, indicating limited antibacterial activity and delayed tissue regeneration in the absence of the active drug (lincomycin HCl). The presence of infection further hindered healing efficiency by prolonging the inflammatory phase. Conversely, wounds treated with LS (LCH–CSNPs–NGs infected with *S. aureus*) and LE (LCH–CSNPs–NGs infected with *E. coli*) demonstrated markedly accelerated healing. Noticeable wound contraction and scab reduction were evident from day 5 onward, with almost complete closure observed by day 12. The LS and LE groups exhibited minimal signs of infection, reduced inflammation, and enhanced tissue regeneration, indicating the potent antimicrobial and wound-healing efficacy of lincomycin HCl-loaded chitosan nanoparticles incorporated in nanogels.

**Fig. 10:**
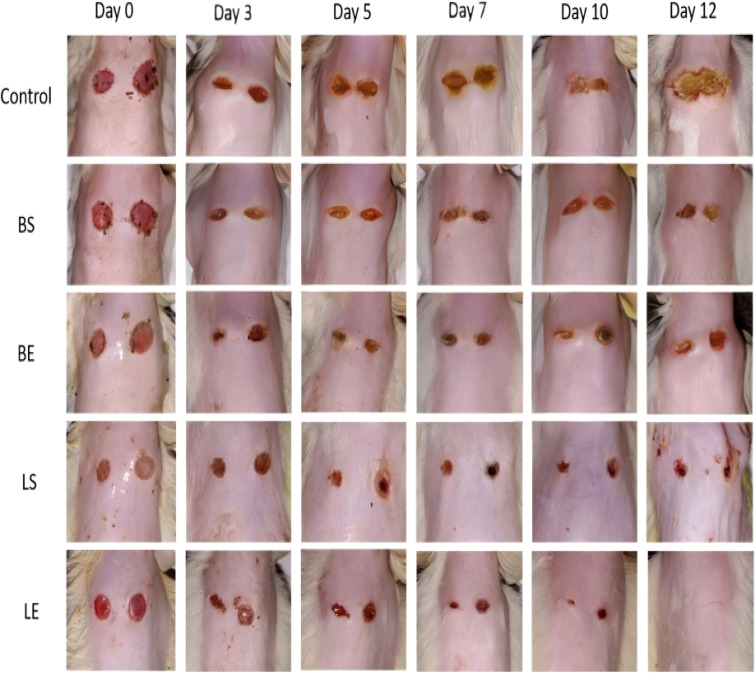
Representative images showing the wound healing progression in different treatment groups over 12 days: control (untreated), BS (blank CSNPs infected with *S. aureus*), BE (blank CSNPs infected with *E. coli*), LS (LCH–CSNPs–NGs infected with *S. aureus*), and LE (LCH–CSNPs–NGs infected with *E. coli*). Images captured on days 0, 3, 5, 7, 10, and 12 show comparative healing efficiency.

The superior healing outcomes in the LS and LE groups can be attributed to the synergistic effects of chitosan, which promotes hemostasis and tissue regeneration, and lincomycin HCl, which provides effective antibacterial activity against both *S. aureus* and *E. coli*. Moreover, the sustained drug release from the nanogel matrix likely maintained a localized therapeutic concentration at the wound site, reducing microbial load and facilitating continuous tissue repair. Overall, the visual progression in this figure supports the hypothesis that LCH–CSNPs–NGs significantly accelerate wound closure and enhance antibacterial protection compared to blank and untreated controls. These results confirm the potential of lincomycin HCl-loaded chitosan nanogels as an effective topical therapy for infected wound management^42,43^ as depicted in Fig. 10.

## 4. Conclusion

This study successfully formulated and characterized LCH–CSNPs that were further developed into a nanogel system suitable for topical wound healing. The nanoparticles exhibited desirable physicochemical properties, sustained drug release, and enhanced antibacterial activity. Rheological behavior and *in vivo* wound contraction studies further confirmed the suitability and therapeutic potential of the nanogel formulation. The non-toxic, biocompatible nature of the system, along with its ability to accelerate tissue regeneration, highlights its promise as a novel wound management strategy. Future work may explore clinical translation and scale-up for commercial topical formulations.

## Author contributions

Aisha Sethi: conceptualization, supervision, project administration, investigation; Rabia Zaheer: conceptualization, methodology, formal analysis, writing – original draft, data curation, investigation; Shazia Akram Ghumman: formal analysis, methodology, writing – review & editing; Asif Massud: software analysis, data curation, visualization; writing, review & editing; Shazia Noureen: data curation, visualization; review & editing; Ali Irfan: formal analysis, funding acquisition, visualization, writing – review & editing; Mahwish Arshad: validation, resources, methodology, data curation, visualization, writing – review & editing; Muhammad Khawar Abbas: data curation; investigation, writing – review & editing; Mudassar Mazher: formal analysis, investigation, writing – review & editing; Yousef A. Bin Jardan: funding acquisition, formal analysis, visualization; project administration, writing – review & editing. All authors have read and agreed to the published version of the manuscript.

## Conflicts of interest

The authors have no conflicts of interest to declare.

## Funding

This research is funded by the Ongoing Research Funding Program (ORF-2026-457), King Saud University, Riyadh, Saudi Arabia.

## Data Availability

All the data of this study are contained in the manuscript. For any additional data or information needed regarding this research, please contact the corresponding author, Aisha Sethi at ayeshasethi786@gcuf.edu.com (A.S). The preprint is already submitted at preprint forum at link: https://www.preprints.org/manuscript/202502.2311/v1.

## References

[cit1] Adami R., Liparoti S., Della Porta G., Del Gaudio P., Reverchon E. (2017). Lincomycin hydrochloride loaded albumin microspheres for controlled drug release, produced by Supercritical Assisted Atomization. J. Supercrit. Fluids.

[cit2] Menninger J. R. (1995). Mechanism of inhibition of protein synthesis by macrolide and lincosamide antibiotics. J. Basic Clin. Physiol. Pharmacol..

[cit3] Kaladhar D., Ramakrishna A. V. (2013). Molecular Analysis of Streptococcus thermophilus Supporting Probiotic Action towards Drug Targets. Asian J. Pharm. Res. Health Care.

[cit4] Ning Z. H. (2015). *et al.*, Effect of exposure routes on the relationships of lethal toxicity to rats from oral, intravenous, intraperitoneal and intramuscular routes. Regul. Toxicol. Pharmacol..

[cit5] Xu Y. (2023). *et al.*, Nanomaterial-based drug delivery systems for pain treatment and relief: from the delivery of a single drug to Co-delivery of multiple therapeutics. Pharmaceutics.

[cit6] Nanda A., Nanda S., Khan Ghilzai N. (2006). Current developments using emerging transdermal technologies in physical enhancement methods. Curr. Drug Delivery.

[cit7] Mohd Zaid N. A. (2023). *et al.*, Promising natural products in new drug design, development, and therapy for skin disorders: An overview of scientific evidence and understanding their mechanism of action. Drug Des., Dev. Ther..

[cit8] Skwarczynski M. (2022). *et al.*, Antimicrobial activity enhancers: Towards smart delivery of antimicrobial agents. Antibiotics.

[cit9] Mallah S. Z. (2025). *et al.*, Antibacterial Bilayer Hydrogel Dressing for Effective Wound Management: Synthesis and Characterization. BioNanoScience.

[cit10] Yao J. (2023). *et al.*, Recent advances in strategies to combat bacterial drug resistance: antimicrobial materials and drug delivery systems. Pharmaceutics.

[cit11] Alehosseini E., Shahiri Tabarestani H., Kharazmi M. S., Jafari S. M. (2022). Physicochemical, thermal, and morphological properties of chitosan nanoparticles produced by ionic gelation. Foods.

[cit12] Shirolkar M. M. (2021). *et al.*, Antibiotics functionalization intervened morphological, chemical and electronic modifications in chitosan nanoparticles. Nano-Struct. Nano-Objects.

[cit13] Popescu-Pelin G. (2018). *et al.*, Lincomycin–embedded PANI–based coatings for biomedical applications. Appl. Surf. Sci..

[cit14] Leyva-Porras C. (2019). *et al.*, Application of differential scanning calorimetry (DSC) and modulated differential scanning calorimetry (MDSC) in food and drug industries. Polymers.

[cit15] Hadidi M., Pouramin S., Adinepour F., Haghani S., Jafari S. M. (2020). Chitosan nanoparticles loaded with clove essential oil: Characterization, antioxidant and antibacterial activities. Carbohydr. Polym..

[cit16] Dong W. (2020). *et al.*, Self-assembled lecithin/chitosan nanoparticles based on phospholipid complex: a feasible strategy to improve entrapment efficiency and transdermal delivery of poorly lipophilic drug. Int. J. Nanomed..

[cit17] Zielińska A. (2020). *et al.*, Polymeric nanoparticles: production, characterization, toxicology and ecotoxicology. Molecules.

[cit18] Ghumman S. A. (2023). *et al.*, Mimosa pudica mucilage nanoparticles of losartan potassium: Characterization and pharmacodynamics evaluation. Saudi Pharm. J..

[cit19] El-Hadedy D., El-Nour S. A. (2012). Identification of Staphylococcus aureus and Escherichia coli isolated from Egyptian food by conventional and molecular methods. J. Genet. Eng. Biotechnol..

[cit20] Weng J., Tong H. H., Chow S. F. (2020). In vitro release study of the polymeric drug nanoparticles: development and validation of a novel method. Pharmaceutics.

[cit21] Saleem S. (2023). *et al.*, Toxicity profiling of Burgmansia aurea Lagerh. Leaves using acute and sub-acute toxicity studies in rats. J. Ethnopharmacol..

[cit22] Pervaiz F., Mushtaq R., Noreen S. (2021). Formulation and optimization of terbinafine HCl loaded chitosan/xanthan gum nanoparticles containing gel: Ex-vivo permeation and in-vivo antifungal studies. J. Drug Delivery Sci. Technol..

[cit23] Ahmed K. A. A. (2024). *et al.*, Cumin (Cuminum cyminum L.) seeds accelerates wound healing in rats: possible molecular mechanisms. Sking Res. Technol..

[cit24] Sethi A., Ahmad M., Huma T., Khalid I., Ahmad I. (2021). Evaluation of low molecular weight cross linked chitosan nanoparticles, to enhance the bioavailability of 5-flourouracil. Dose-Response.

[cit25] Bhatia S. (2024). *et al.*, Tuning the structure and physiochemical properties of sodium alginate and chitosan composite films through sodium tripolyphosphate (STPP) crosslinking. Int. J. Biol. Macromol..

[cit26] Divya K., Vijayan S., George T. K., Jisha M. (2017). Antimicrobial properties of chitosan nanoparticles: Mode of action and factors affecting activity. Fibers Polym..

[cit27] Qing X. (2021). *et al.*, Preparation and properties of polyvinyl alcohol/N-succinyl chitosan/lincomycin composite antibacterial hydrogels for wound dressing. Carbohydr. Polym..

[cit28] Li H., Wu J., Bai J., Wu J., Wu J. (2023). Determination of lincomycin in milk using Cu-based metal-organic framework adsorbent and liquid chromatography-tandem mass spectrometry. Molecules.

[cit29] Benamer Oudih S. (2023). *et al.*, Chitosan nanoparticles with controlled size and zeta potential. Polym. Eng. Sci..

[cit30] Lemos P. V. F. (2020). *et al.*, Preparation and characterization of C-phycocyanin coated with STMP/STPP cross-linked starches from different botanical sources. Int. J. Biol. Macromol..

[cit31] Mdzinarashvili T., Khvedelidze M., Shekiladze E., Koenneke A., Schneider M. (2020). Stability of various PLGA and lipid nanoparticles in temperature and in time and new technology for the preparation of liposomes for anticancer and antibiotic loading. J. Therm. Anal. Calorim..

[cit32] Cai T. (2019). *et al.*, Preparation of monodisperse magnetic surface molecularly imprinted polymers for selective recognition of lincomycin hydrochloride in milk. J. Liq. Chromatogr. Relat. Technol..

[cit33] Noreen S. (2022). *et al.*, pH responsive Abelmoschus esculentus mucilage and administration of methotrexate: in-vitro antitumor and in-vivo toxicity evaluation. Int. J. Mol. Sci..

[cit34] Qubtia M. (2024). *et al.*, Evaluation of plant-based silver nanoparticles for antioxidant activity and promising wound-healing applications. ACS Omega.

[cit35] Abdul-Jabbar A. M. (2022). *et al.*, Combined anti-bacterial actions of lincomycin and freshly prepared silver nanoparticles: overcoming the resistance to antibiotics and enhancement of the bioactivity. Antibiotics.

[cit36] Weldrick P. J., Hardman M. J., Paunov V. N. (2019). Enhanced clearing of wound-related pathogenic bacterial biofilms using protease-functionalized antibiotic nanocarriers. ACS Appl. Mater. Interfaces.

[cit37] Ganjoo R., Soni S., Ram V., Verma A. (2016). Medium molecular weight chitosan as a carrier for delivery of lincomycin hydrochloride from intra-pocket dental film: Design, development, in vitro and ex vivo characterization. J. Appl. Pharm. Sci..

[cit38] Hirai Y. (2021). *et al.*, Characterization of compound A, a novel lincomycin derivative active against methicillin-resistant Staphylococcus aureus. J. Antibiot..

[cit39] IF K., Onwuliri A., Ehinmidu J., Oladosu P. (2020). Evaluation of the Antibacterial Activities, Acute Toxicity and Immuno-stimulatory Potential of Adenodolichos paniculatus Chloroform root. Extract.

[cit40] Panonnummal R., Sabitha M. (2018). Anti-psoriatic and toxicity evaluation of methotrexate loaded chitin nanogel in imiquimod induced mice model. Int. J. Biol. Macromol..

[cit41] Khalid Q., Ahmad M., Usman Minhas M. (2018). Hydroxypropyl-β-cyclodextrin hybrid nanogels as nano-drug delivery carriers to enhance the solubility of dexibuprofen: Characterization, in vitro release, and acute oral toxicity studies. Adv. Polym. Technol..

[cit42] Kesharwani P., Jain A., Srivastava A. K., Keshari M. K. (2020). Systematic development and characterization of curcumin-loaded nanogel for topical application. Drug Dev. Ind. Pharm..

[cit43] Kaur L., Jain S. K., Singh K. (2015). Vitamin E TPGS based nanogel for the skin targeting of high molecular weight anti-fungal drug: development and in vitro and in vivo assessment. RSC Adv..

